# Nano-Enabled Agrochemicals for Heavy Metal Remediation in Agriculture: Current Status, Mechanisms, and Future Prospects

**DOI:** 10.3390/nano15201588

**Published:** 2025-10-17

**Authors:** Muhammad Mudassir Nazir, Guanlin Li, Mohsin Nawaz, Temoor Ahmed, Muhammad Noman, Sanaullah Jalil, Xiaojun Zheng, Xunfeng Chen, Daolin Du

**Affiliations:** 1 Key Laboratory of Zhenjiang, School of Environment and Safety Engineering, Jiangsu University, Zhenjiang 212013, China; dr.mudasar@ujs.edu.cn (M.M.N.); mna@ujs.edu.cn (M.N.); xjzheng@ujs.edu.cn (X.Z.); 2 Institute of Biotechnology, Zhejiang University, Hangzhou 310058, China; temoorahmed@zju.edu.cn (T.A.); sanaullahjalil@zju.edu.cn (S.J.); 3 Department of Life Sciences, Western Caspian University, Baku AZ1001, Azerbaijan; 4 Institute of Plant Protection and Microbiology, Zhejiang Academy of Agricultural Sciences, Hangzhou 310021, China; m.noman@zju.edu.cn; 5 Department of Plant Biotechnology, Korea University, Seoul 02481, Republic of Korea; 6 Jingjiang College, Jiangsu University, Zhenjiang 212013, China

**Keywords:** heavy metals, mineral nutrition, nano-agrochemicals, plant stress tolerance, sustainable agriculture

## Abstract

Heavy metals (HMs) contamination in agricultural soils poses significant risks to crop production and human health through bioaccumulation in the food chain. While traditional remediation techniques exist, they often face limitations including high operational costs, low efficiency, and time-intensive processes. Nano-enabled agrochemicals have emerged as a promising solution for HM remediation in contaminated soils. In this review, we highlight distinct nano-enabled mechanisms involved in HMs remediation in agricultural soils. Further, this review describes HM remediation potential of three different classes of nano-agrochemicals exhibiting unique physicochemical properties, such as surface charge, controlled release capability, and metal chelating ability, etc. Nano-agrochemicals also enhance plant resilience through multiple pathways, such as the regulation of nutrient profiles and photosynthesis, activation of antioxidant defense systems, modulation of protein and osmolyte synthesis, stimulation of phytohormone pathways, and activation of stress-responsive transcription factors. While nano-agrochemicals show tremendous potential for sustainable agriculture, their environmental impact and safety considerations require careful assessment. The review highlights the need for continued research to fully understand nano-agrochemical interactions with plants and soil ecosystems, and to develop improved strategies for their safe and effective implementation in agricultural systems. Future studies should focus on optimizing nano-agrochemical formulations, investigating long-term effects, and establishing comprehensive risk assessment frameworks.

## 1. Introduction

The release of HMs into agricultural soils via natural processes (soil formation, rock weathering) as well as anthropogenic (human-induced) activities like industrialization and transportation, use of extensive fertilizers and pesticide, poses a serious threat to crop production and human health due to bioaccumulation within the food chain [1,2]. This threat is amplified by the bioaccumulation of HMs within the food chain [3]. Due to their non-degradable and persistent nature over geological timescales, HMs are considered as destructive contaminants for all ecosystems [4] and represent a global environmental threat [5]. The ecological risks of HMs are closely connected to their dispersion in groundwater resources and accelerating mobility in soil environments like soil rhizosphere [6]. Most prevalent HMs ions in industrial effluents include copper (Cu), lead (Pb), chromium (Cr), cadmium (Cd), and nickel (Ni) [7]. These HMs can be easily absorbed by roots, transferred to above-ground parts and induce various physiological, biochemical, and transcriptional changes in plants [8]. The accumulation of these HMs in plants causes severe impacts on plant cells by inducing oxidative stress through the generation of reactive oxygen species (ROS), leading to DNA damages, proteins denaturation, and imbalanced cell homeostasis, which finally leads to plant death [9,10]. Furthermore, the entry of these HMs into the food chain through cereal crops compromises agricultural yield, product quality, and human health [11].

In the past decades, several on-site/off-site remediation techniques including vitrification, soil flushing, electrokinetic extraction, and bioremediation have been adopted to immobilize or remove HMs from soil, thereby improving soil health and quality for the cultivation of healthy cash crops [11,12]. While advanced strategies like microfluidics show promise for the precise detection and separation of HMs in controlled settings [13], their practical application is often limited to lab-scale analysis due to their inherently low throughput [14]. For large-scale field remediation, on-site techniques are generally preferred to manage the massive volume of contaminated soil in situ, thereby avoiding the prohibitive costs and logistical challenges of excavation and transport [12]. However, the adoption of these techniques may lead to high operational cost, low efficiency, time-consuming processes, and lower degradability of HMs on spatial and temporal scales under actual field conditions [15]. Hence, there is an urgent need for novel, eco-friendly, cost effective, nontoxic, and recyclable adsorbents for large-scale remediation of HMs-contaminated soils [16].

Nano-remediation has recently emerged as a promising strategy to overcome these challenges. The application of nano-agrochemicals, such as nanoparticles (NPs) and nano-fertilizers (NFs), can facilitate soil remediation while simultaneously enhancing crop health and agricultural productivity [12]. Nano-agrochemicals can improve physiochemical and biological properties of soil, enhance soil fertility, plant growth, and increase crop yield and quality, all while minimizing operational costs and environmental impacts [16]. Their efficacy stems from unique properties such as large surface area, specific shape, smaller size, high reactivity, and surface functional groups, which enable them to effectively immobilize toxic HMs from agricultural soil [15]. Additionally, they possess various mechanisms to reduce oxidative stress in plants grown in contaminated soils, including the nano-delivery of nutrients, the direct ROS-scavenging by nanozymes [12], and the triggering of stress-responsive genetic mechanisms.

For instance, the application of ZnO-NPs (100 mg kg^−1^) has been shown to immobilize Cd in soil via direct adsorption, reducing its concentration in roots (32%), shoots (20%), husks (36%), and grains (26%) of wheat (*Triticum aestivum* L.) [17]. However, the stability and efficacy of such metal-based nano-agrochemicals are highly dependent on soil conditions [18]. Specifically, the dissolution of ZnO NPs, particularly under acidic soil conditions, can release Zn^2+^ ions, potentially leading to phytotoxicity and altering soil microbial communities [19]. In comparison, Si-NPs (100%) immobilized arsenic (As) through complexation, reducing both plant uptake and oxidative stress up to 70% by improving the antioxidative system in maize (*Zea mays* L.) [19]. Therefore, nano-agrochemicals present a superior and sustainable alternative due to their long-term effectiveness with lower operational costs, offering a better solution for HM immobilization of HMs in contaminated soils compared to other traditional methods. A deeper understanding of the tripartite interactions among plants, nano-agrochemicals, and soil ecosystems is crucial to optimizing their application and ensuring the safe, effective remediation of contaminated environments.

### Scope and Methodology of the Review

**Figure 1:**
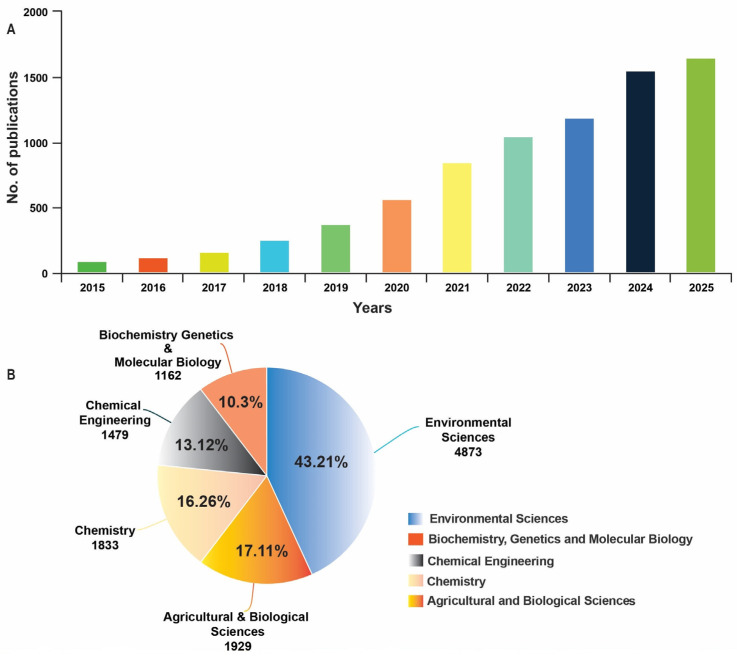
Summary of papers by using keywords such as “nanoparticles,” “nano-agrochemicals,” “heavy metal remediation,” “soil contamination,” and “plant stress tolerance.” (**A**) Number of papers published between 2015 and 2025; (**B**) publication in different areas of research. Data was accessed on 10 October 2025.

This review comprehensively examines the latest developments in nano-enabled agrochemicals for remediating HMs in agricultural soils. It systematically analyzes the remediation mechanisms of three primary classes, metal oxide, carbon-based, and polymeric nanocomposites, within soil–plant systems. The physiological and molecular responses of plants to these nano-agrochemicals under HM stress are also evaluated. Finally, the review critically assesses the associated environmental impacts and safety considerations, providing a balanced perspective on both the remediation potential and the risks of nano-agrochemicals for sustainable agriculture. To compile this comprehensive review, an extensive literature search was conducted using major academic databases including Scopus, Web of Science, and Google Scholar. The search encompassed research articles published between 2015 and 2025, utilizing keywords such as “nanoparticles,” “nano-agrochemicals,” “heavy metal remediation,” “soil contamination,” and “plant stress tolerance”, Figure 1. Relevant studies were selected based on their focus on mechanistic insights, remediation efficacy, and environmental safety assessments of nano-enabled strategies for HM remediation in agricultural systems.

## 2. Types of Nano-Enabled Agrochemicals for Heavy Metal Remediation

Nanotechnology, especially nano-enabled agrochemicals, has emerged as a novel and effective strategy for enhancing food security by boosting crop production and mitigating abiotic stresses including HMs contamination [20]. In this context, NPs are defined as engineered materials with at least one external dimension in the nanoscale range of approximately 1–100 nm, a size regime that confers the unique physicochemical properties central to their function [21]. These properties allow for the tailored synthesis of nano-agrochemicals with various sizes, geometries, and functionalities to address agricultural needs and remediate HMs in contaminated soils [4].

Metal oxide nanomaterials (MONs) are a prominent class of nano-agrochemicals that can be easily synthesized via top-down (physical) or bottom-up (chemical) approaches [21]. The bottom-up approach, which involves the oxidation or reduction of metal salt precursors, is commonly employed as it provides precise control over particle size within the nanoscale range [22]. Numerous methods have been employed to synthesize metal oxide nanomaterials, including lithography, co-precipitation, inert gas condensation, and milling techniques. According to Inobeme et al. [23], over 1300 commercial NPs have been employed in a variety of applications, including agriculture. The following section will provide details about types of NPs and their implications in soil for HMs remediations in soil–plant systems.

### 2.1. Metal Oxide Nanomaterials

**Figure 2:**
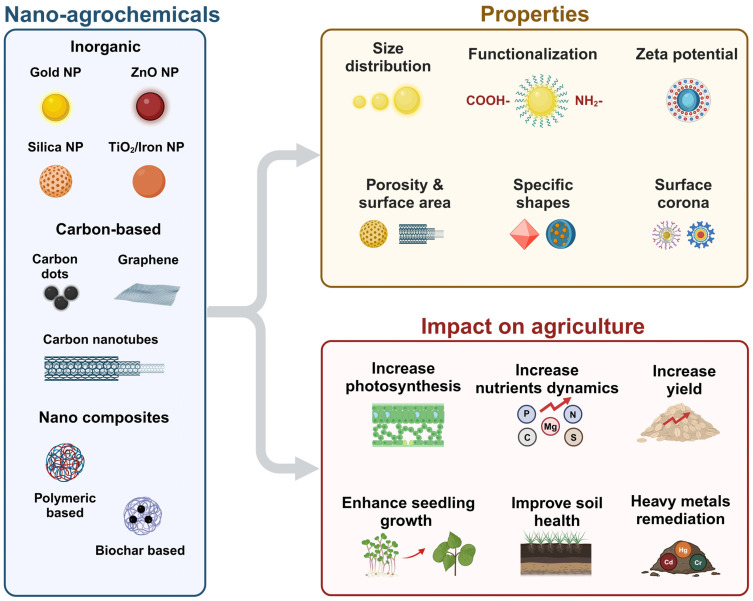
Schematic representation of nano-agrochemical types, their characteristics, and applications in agriculture.

Metal oxide nanomaterials (MONs) are considered as most promising candidates in diverse areas of chemistry, materials science, biotechnology, and environmental remediation [24,25]. Specific MONs such as copper oxides (CuO, Cu_2_O), iron oxide (FeO), selenium oxide (SeO), zinc oxide (ZnO), magnesium oxide (MgO), nickel oxide (NiO), and indium oxide (In_2_O_3_) are particularly valuable as sensing materials due to their high sensitivity, quick response/recovery times, excellent reproducibility, stability, and cost-effectiveness [4]. The distinct surface, thermal, electrical, and physical properties of MONs, which differ from their bulk counterparts, underpin their reactivity. This reactivity with biomolecules is governed by a range of factors, including particle size, core composition, shape, surface properties, purity, stability, and the manufacturing procedure Figure 2 [26].

In agriculture, NPs such as FeO, ZnO, and MgO have been widely used due to their ability to deliver nutrients and their exceptional properties, including high surface energy, improved surface area, quantum confinement, and catalytic and sorption capabilities, which are effective for immobilizing HMs in contaminated soils [4]. For instance, nanoscale zero-valent iron (nZVI) possesses a large specific surface area and numerous adsorption sites, and can reduce HM ions to less toxic or insoluble forms in soil [27]. A key example is the conversion of highly toxic hexavalent chromium (Cr^6+^) to its less toxic trivalent form (Cr^3+^), thereby reducing its mobility in the soil system [27,28]. However, the dissolution of ZnO NPs in acidic soils can release Zn^2+^ ions, raising concerns about phytotoxicity and harm to soil microbes [19]. The remediation mechanisms of metal oxide nano-agrochemicals are diverse, encompassing adsorption, ion exchange, reduction, precipitation, catalytic degradation, and stabilization, all of which interact with HMs in soil to reduce their phytoavailability. This is demonstrated by a field experiment where the application of nZVI (3% *w*/*w*) immobilized Zn, Cd, As, Cu, Pb by 65%, 72%, 74%, 95%, and 66%, respectively, over 60 days, through complexation, precipitation, and direct adsorption [29].

The interactions between NPs and HMs in the soil matrix are governed by a complex suite of interfacial forces [30]. These include van der Waals forces (e.g., dipole–dipole interactions), electrostatic interactions (influenced by the ionic strength and pH of the soil solution), and Lewis acid–base interactions [31]. Furthermore, hydrophobic interactions and electrosteric stabilization, imparted by coatings of natural organic matter on NP surfaces, play a critical role in determining the aggregation, mobility, and ultimate reactivity of NPs, thereby controlling the immobilization and bioavailability of HMs for plant roots [23].

MONs stabilize HMs primarily by forming complexes with metal ions, which limits leaching, immobilizes them in soil, and reduces their uptake and translocation in plants [4]. Wang et al. [32] demonstrated that applying nano-silica (1% *w*/*w*) reduced the Cd leachability by 36% and bioavailability by 54%, transforming it into more stable fractions. This treatment also increased grains yield by 33%, and Si contents in leaf, stem, and husk of wheat plants by 55%, 50%, and 37%, respectively. The competition between silicon and Cd for similar uptake pathway is a probable mechanism for reduced absorption and translocation of Cd in *Triticum aestivum* plants [33]. Similarly, Manzoor et al. [34] demonstrated that Cd was immobilized by FeO-NPs, due to their higher reactivity, large surface area, and electrostatic attraction, which subsequently reduced its translocation into the plant system. In addition, nano-enabled nutrient delivery systems supply plants with essential nutrients like Fe, Mg, Zn, and Ca, which compete with Cd^2+^ for uptake, thereby reducing Cd^2+^ concentration within plant tissues and regulating key metabolic processes to improve growth and development [34].

### 2.2. Carbon Nanomaterials

Carbon (C)-based nano-agrochemicals are a promising option for remediation of HMs in contaminated soils, due to their unique properties such as larger surface area, high porosity, low density, mechanical strength, hollow structures, thermal/electrical conductivity, and superior sorption capacity [24,26,35]. The impact of these materials on soil properties can vary; for instance, black carbon nanoparticles were found to reduce soil pH more significantly in post-harvest ryegrass than in a leaf red beet crop [36]. Among C-based nanomaterials, carbon dots (CDs) exhibit unique properties, such as greater biocompatibility, rich surface chemistry, and low cytotoxicity, making them potential candidates for agricultural applications [26,37]. For example, N-doped CDs (0.2 mg mL^−1^) markedly increased the growth rate of mung bean plants by 200% [38]. Similarly, CDs application improved seed germination, enhanced photosynthesis and Rubisco enzyme activity by 42%, and increased rice yield by 14% [39].

Carbon NPs can enhance soil health by stimulating root exudation and promoting the growth of phyto-beneficial microbes [40]. Their nutrient-holding capacity is attributed to their microporous structure, which facilitates the absorption and controlled release of essential micro- and macronutrients [41]. Carbon nanotubes (CNTs), including single-walled (SWCNTs) and multi-walled (MWCNTs) variants, have been widely studied for their interactions with HMs [42]. They have been shown to improve seed germination and, when loaded with nutrients (N, P, K), can function as effective slow-release nano-fertilizers in the agricultural system [43]. For instance, in corn (*Zea mays* L.), the application of carbon NPs (100 mg L^−1^) increased seed germination and seedling growth while reducing Cu^2+^ uptake and associated toxicity [44]. The application of MWCNTs (500 mg kg^−1^) immobilized As (8.19%) and Cd (16.29%) in soil, enhanced micronutrient uptake and antioxidative defense system, promoted plant growth, while reducing the oxidative stress in *Solanum nigrum* L. grown in multi-contaminated soil [9].

While CNTs show significant benefits, their potential phytotoxicity requires consideration. For instance, CNTs have been reported to delay flowering time in rice by one month [39]. Conversely, they can also induce positive stress responses, such as rapidly increasing peroxidase activity, phytohormone levels, and the expression of stress-responsive proteins in tomato plants [45]. This ability to modulate stress-responsive gene expression makes CNTs valuable for sustainable crop management in challenging environments [41].

### 2.3. Polymeric Nanocomposites

Polymeric nanocomposites are sophisticated materials created by incorporating nanoscale fillers such as carbon nanotubes (CNTs), graphene, nano-clays, or metal oxide nanoparticles into a polymer matrix [46]. This integration results in enhanced physical, chemical, and mechanical properties, making them ideal for advanced environmental and agricultural applications [24,37].

Recently, polymer-based nanoparticles have appeared as a safe solution in agricultural practices for the targeted delivery of nutrients and the remediation of HMs, while preserving soil health [35]. A key function of these nanomaterials is to protect active agrochemical ingredients from premature degradation and control their release, thereby improving efficiency and reducing environmental impact [24,47]. For instance, Giroto et al. [48] developed a nanocomposite consisting of a urea-loaded thermoplastic starch matrix infused with hydroxyapatite nanoparticles (nHAP). This system simultaneously regulates the release of nitrogen from urea and phosphorus from the nHAP, significantly improving nutrient use efficiency. Furthermore, the large surface area of the nHAP composite enhances its capacity to adsorb and bind HM ions, effectively immobilizing them in the soil and reducing their phytoavailability [18]. This dual functionality was demonstrated in a study where nHAP application (5 g kg^−1^) increased soil pH, released phosphorus, immobilized lead through complex formation, and decreased its absorption in ryegrass (*Lolium perenne* L.) [49].

There is a growing research focus on the use of natural polymers, such as polysaccharides, for these applications [35]. A substantial advantage of natural polymers is their biodegradability by microbes, which results in environmentally benign products, unlike non-biodegradable synthetic polymers [35]. Overall, the encapsulation of agrochemicals within polymeric nanocomposites enhances material integrity, biocompatibility, and nutrient use efficiency [50]. This approach not only boosts agricultural sustainability but also opens new research avenues for developing effective alternatives that minimize ecotoxicity [46].

## 3. Mechanisms of Heavy Metal Remediation

The accumulation of HMs at toxic levels hinders the uptake of essential mineral nutrients, disturbs ion homeostasis, and induces oxidative stress by impairing essential cellular processes such as photosynthesis and respiration [11]. This ultimately leads to damage of vital biomolecules, including lipids, proteins, and DNA [8]. Nano-agrochemicals mitigate these harmful effects primarily by reducing HM absorption and translocation in plants. This action helps restore ionic balance and alleviates oxidative stress, partly through the accumulation of protective osmolytes such as proline, glycine betaine, and soluble sugars [51].

**Figure 3:**
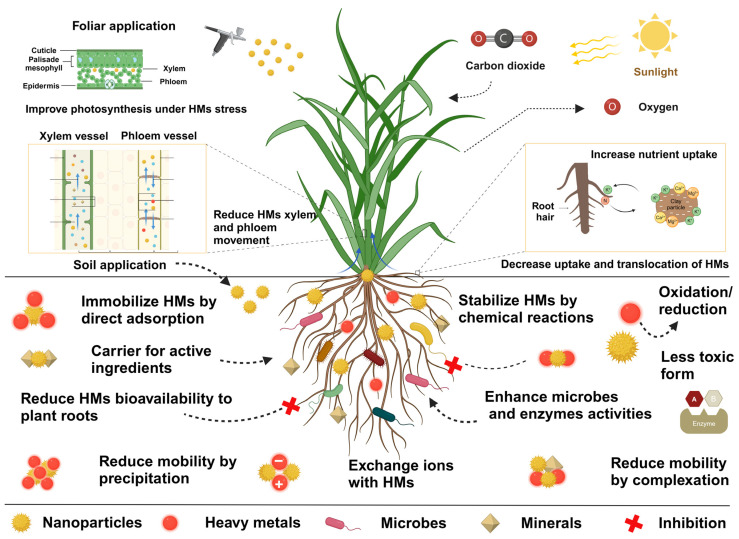
Schematic illustration of the interaction between nano-agrochemicals and heavy metals in soil–plant systems. Nano-agrochemicals enhance soil properties by promoting the immobilization of HMs, thereby preventing their uptake and radial transport in plants and minimizing environmental toxicity. Additionally, NPs can influence soil microbial communities and facilitate the detoxification of HMs, contributing to improved soil health, fertility, and ecosystem stability.

In contaminated soils, nano-agrochemicals possess several mechanisms including adsorption, immobilization, complexation, precipitation, and redox reactions to reduce the bioavailability and mobility of HMs [20], Figure 3. The efficacy of these processes is strongly influenced by soil physicochemical properties, such as structure, texture, clay mineral content, pH, cation exchange capacity (CEC), organic matter, and the native microbial population, all of which govern the dispersion, stability, and transport of both nanomaterials and HMs [4].

The potential of metallic nano-fertilizers for remediating toxic heavy metal(loid)s has been widely documented [20,26]. Their application can directly alter soil conditions to immobilize HMs; for instance, by raising soil pH, which can prompt HMs like Zn, Pb, and Cd to precipitate as less soluble hydroxides or carbonates, thereby reducing their bioavailability [22]. The addition of CuO and ZnO NPs has been shown to increase soil pH and shift HM distribution into less bioavailable fractions. However, the dissolution of ZnO NPs under acidic conditions may elevate Zn^2+^ bioavailability, raising potential phytotoxicity concerns [19]. In contrast, TiO_2_ NPs reduce Cd bioavailability by facilitating its conversion into less soluble forms, rather than through pH alteration [10]. The following sections will detail the mechanisms of HM immobilization and detoxification mediated by the exogenous application of NFs at the soil–plant interface.

### 3.1. Immobilization of Heavy Metals in Soil–Plant Systems

Nano-fertilizers present a promising approach to immobilize HMs in soil–plant systems, leveraging their unique physicochemical properties including small size, high stability, chemical reactivity, and specific large surface area [35]. Several studies have confirmed that metal oxide NFs can effectively immobilize HMs in soils and reduce their phytoavailability through various mechanisms [18,52]. A primary mechanism is direct absorption, where NFs facilitate HMs remediation through redox reactions in contaminated soils [18]. For instance, the addition of nZVI (20 mg kg^−1^) reduced As, Pb, and Hg concentrations by 65%, 54%, and 61%, respectively, within 2 months, in multi-contaminated soil [53]. Similarly, nano-silica (3% *w*/*w*) markedly lowered the bioavailability of Cd, Pb, and As by 80%, 97%, and 85%, respectively [54]. Soil amendment with nano-silica (SiO_2_-SH; 4% *w*/*w*) significantly decreased the absorption of Cu, Cd, and Pb in lettuce by 5%, 89%, and 43%, respectively, and in pak choi by 76%, 92%, and 68%, respectively [55]. This immobilization occurred as the SiO_2_-SH transformed the metals from acid-soluble forms into less bioavailable, oxidizable, and reducible fractions, thereby limiting their extractability from the soil.

The efficacy of nano-remediation extends across various plant systems, as evidenced by nZVI application (1% *w*/*w*), decreasing As concentration in the rhizosphere by 80% and reducing its accumulation in roots (47%) and shoots (24%) of *Helianthus annuus* L. [56]. In wheat, nano-Fe_3_O_4_ (2000 mg L^−1^) markedly reduced the uptake of Zn, Pb, Cd, and Cu by 23%, 54%, 65%, and 68% in roots, and in shoots by 11%, 17%, 99%, and 52%, respectively [57]. Furthermore, FeO-NPs (100 mg kg^−1^) decreased Cd bioavailability in soil by 69%, while reducing root absorption, shoot accumulation, and acropetal translocation in wheat by 38%, 72%, and 54%, respectively [34]. The reduced phytoavailability of HMs is closely associated with chemical interactions and surface functional groups (e.g., -OH, -NH_2_, -SH, -COOH) on NFs, which provide active sites for HM ion exchange [18,52].

The inclusion of NFs composites further enhances remediation capabilities through improved properties such as CEC, higher surface-to-volume ratio, improved stability, mechanical strength, and enhanced thermal/electrical conductivity [35,52]. The addition of nano-hydroxyapatite (nHAP: 5 gkg^−1^) decreased mobility of Pb in soil and reduced the uptake in ryegrass (*Lolium perenne* L.) plants [49]. Moreover, nHAP increased soil pH, released phosphates and enhanced the formation of insoluble pyromorphite-like complexes, thereby decreasing the Pb mobility in soil. Similarly, Fe_3_O_4_-biochar (Fe-BC) nanocomposites (0.2, and 0.4%) enhanced soil CEC and reduced availability of Cd (6 and 25%) in soil. Additionally, Fe-BC nanocomposites form Fe plaque on roots, which act as a barrier and reduce the Cd uptake, and accumulation in *Oryza sativa* L. [58]. The multifunctional nature of these composites is further demonstrated by nZVI-BC systems, which simultaneously immobilize As and Cd in soil, reducing their uptake via Fe plaque and grain accumulation by 61% and 93%, respectively, in rice plants [59].

The immobilization of HMs by nZVI-BC composites and NFs primarily involves three different mechanisms: (1) adsorption, (2) direct/ortho reduction, and (3) electrostatic attractions [60]. Collectively, nano-agrochemicals employ diverse strategies including adsorption, immobilization, chelation, root barrier enhancement, and nanocarrier delivery of essential nutrients (e.g., Fe, Mg, Zn, Ca) to reduce HM mobility in soil–plant systems and promote sustainable crop production, as summarized in Figure 3.

### 3.2. Mechanisms of Uptake and Translocation

Nano-agrochemicals have potential to reduce the uptake and transport of HMs by direct fixation, adsorption, and immobilization, thereby reducing their phytoavailability to plant roots [18]. One primary mechanism involves the fortification of root vascular systems, where NPs can increase the formation of Casparian strips and suberin lamellae, which act as apoplastic barriers to the radial transport of HMs [61]. For instance, the application of CeO_2_-NPs (500 mg L^−1^) upregulated the expressions of genes (*SaCASP*, *SaGPAT5*, *SaKCS20*, and *SaCYP86A1*) involved in the formation of Casparian strips and suberin and reduced apoplastic movement of Cd by 37% in *Sedum alfredii* [61]. Similar results of reduced Cd uptake by CeO_2_-NPs have been reported in corn (*Zea mays* L.) and soybean (*Glycine max* L.) seedlings [62,63].

A second, highly effective strategy is the direct regulation of HM transport genes. NPs have the ability to reduce the expressions of HMs uptake and transport-related proteins including NRAMP (Natural Resistance-Associated Macrophage Protein), HMA (Heavy Metal ATPase), and ZIP (Zinc/Iron-regulated transporter-like protein) [51]. For instance, application of nZVI (200 mg L^−1^) downregulated the expressions of Cd transport-related genes (*OsIRT1*, *OsNRAMP5*, and *OsHMA3*) in *Oryza sativa* L. roots, reducing its acropetal translocation [64]. Similarly, nZVI (200 mg L^−1^) application markedly reduced the expression of *YSL2*, *YSL15*, *IRT1*, and *IRT2*, which are involved in Cd uptake and accumulation, and increased the expressions of *OsCAX4* and *OsVIT1*, which contributed to Cd chelation in rice vesicles [65]. The application of SiNPs (1.0 mM) decreased the expression levels of *OsLCT1* and *OsNramp5* involved in Cd uptake, and increased the expressions of *OsHMA3* and *OsLsi1*, promoting vacuolar sequestration of Cd in rice [66]. Furthermore, melatonin gold (Mel-Au) NPs downregulated a broad spectrum of Cd transport-related proteins (*OsIRT1*, *OsIRT2*, *OsLCT1*, *OsHMA2*, *OsHMA3*, *OsNramp1*, and *OsNamp5*) in roots and leaves of *Oryza sativa* L. seedlings [67].

The NPs-mediated strategy of downregulating metal transporters is similarly effective in reducing other HMs uptake and translocation. For instance the addition of Si-NPs (100 mg L^−1^) and TiO_2_ NPs (50 mg L^−1^) reduced the expressions of As transporter proteins (*Lsi1*, *Lsi2*, and *Lsi6*), thereby decreasing As uptake and its translocation to leaves [68]. Similarly, Fe_3_O_4_ NPs (50 mg L^−1^) downregulate As uptake transporters *Lsi1* and *Lsi2* genes in rice, reducing As absorption while simultaneously upregulating iron uptake genes to enhance plant growth [69]. The addition of CaO-NPs (25 mg L^−1^) decreased the expression of As transport-related genes (*HvPHT1;6*, *HvPHT1;4*, *HvPHT1;3*, and *HvPHT1;1*), which was attributed to improved Ca uptake, improved antioxidants, and reduced ROS, As uptake, and acropetal translocation from roots to shoots in barley seedlings [70]. The elevated expressions of *ABC1*, *PCS*, and *GSH1* genes ensured As transportation to the vacuoles [71]. This mechanism kept the plant leaves from oxidative stress and improved photosynthetic activity and growth of rice plants.

### 3.3. Mechanisms of Regulation of Nutritional Profile and Photosynthesis

Macronutrients, including potassium (K), nitrogen (N), phosphorus (P), calcium (Ca), sulfur (S), and magnesium (Mg), and micronutrients such as boron (B), zinc (Zn), and manganese (Mn), are essential for maintaining vital metabolic processes in plants [22]. Certain HMs, like Cu, Pb, and Cd, can compete with these nutrients for absorption sites due to their chemical similarity and shared uptake pathways [72]. Because of enzymatic breakdown, HMs exert potent inhibitory effects on pigment production by replacing chlorophyll molecules, leading to reduced photosynthetic efficiency, chlorosis (yellowing of leaves), and impairment of Calvin cycle enzymes like Rubisco [73].

Various researchers have demonstrated the critical functions of NFs in enhancing plant growth by effectively boosting targeted delivery systems [8,51]. For instance, the addition of SiO_2_-NPs (10 μM L^−1^) increased the uptake of micronutrients (Zn, Mn, Cu, and Fe), and macronutrients (Mg, P, Ca, K) in hydroponically grown *Pisum sativum* L. under Cr(100 µM) stressed conditions [74] (Table 1). NFs facilitate the absorption of vital elements like Fe and Mg, which are necessary for chlorophyll production and the activity of photosynthetic enzymes under HMs stress [4]. Applying NFs (50 mg L^−1^) to *Vigna radiata* aids in preserving the ultrastructure of mitochondria and chloroplasts, supports the synthesis of ATP, cyclic, and linear phosphorylation, and the Calvin cycle, thereby sustaining photosynthetic efficacy [75].

Iron and Si are directly involved in photosynthesis and cellular respiration [22]. Application of Fe-NPs (10 mg L^−1^) and Si-NPs (20 mg L^−1^) increased K^+^ content by 31 and 24%, intercellular CO_2_ concentration by 22 and 7%, and net photosynthetic rate by 9 and 4%, respectively, in *Phaseolus vulgaris* plants subjected to Cd toxicity [76]. Nano-silica can increase Mg intake, which boosts the formation of photosynthetic pigments and CO_2_ assimilation rates [77]. Similarly, Si-NP applied at 300–1200 mg kg^−1^ improved nutritional content and enhanced photosynthetic performance in wheat plants under Cd stress [78,79]. Copper (Cu) and Ca are important for various metabolic functions, including photosynthesis and cellular respiration in plants. Exogenous application of CuNPs (100 mg kg^−1^) in soil increased N (41%), Ca^2+^(87%), P (58%), and Cu (75%) contents, reduced the Cd translocation (49%), and improved growth of *Triticum aestivum* seedlings under Cr stress [80]. Likewise, CaO NPs (25 mM) in hydroponic solution promoted the intake of vital nutrients (Ca, Zn, Fe, Mg) and improved the SPAD (38%), Fv/Fm (16%), net photosynthetic rate (44%), and transpiration rate (61%), while reducing Cd content in barley seedlings [81]. This reduced metal uptake, and translocation is attributed to competition between essential nutrients and metal ions. Another study demonstrated that foliar application of ZnO-NPs (100 mg L^−1^) increased Zn (37%), Fe (31%), and chlorophyll pigment (61%) in hydroponically grown rice under 100μM Cr stress [77]. Similar results of enhanced nutritional profile and photosynthetic efficiency have been reported with ZnO-NPs in As-stressed rice plants [82]. While the nutrient delivery of ZnO-NPs is beneficial, it is important to note that their behavior is influenced by soil conditions. Their dissolution in the rhizosphere, a process accelerated in acidic soils, can be a double-edged sword, providing essential Zn^2+^ but also potentially leading to ionic toxicity if not managed properly [19].

**Figure 4:**
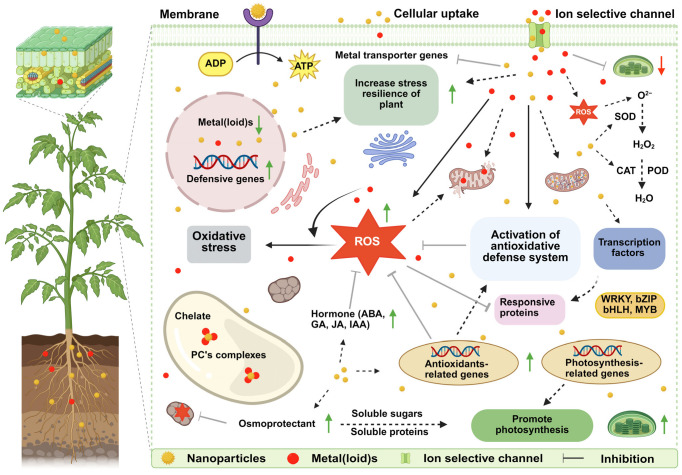
Schematic illustration of nano-agrochemicals-mediated remediation of HMs in plants. Nano-agrochemicals facilitate the reduction in HMs uptake and ameliorate HM-induced toxicity by enhancing the plant’s antioxidant defense system, reducing ROS, and maintaining ions homeostasis. Abbreviations: NP, nanoparticles; HMs, heavy metals; PC, phytochelatins; ROS, reactive oxygen species. This figure was created using BioRender.com.

Beyond nutrient competition, certain nano-agrochemicals directly enhance photosynthetic machinery and its underlying genetic regulation. For instance, TiO_2_ NPs improved the hydrolyzation of water generated by light, stabilizing the electron transport chain and increasing plant photosynthetic efficiency [83]. This can be due to enhanced expression of *LHCII-b* genes in the thylakoid membrane, which increases the assimilation of light in the chloroplast [84]. In another study, application of nano-Se (100 μmol L^−1^) markedly enhanced Rubisco activities, chlorophyll contents photosynthesis-related gene expressions (*RbcL*, *psbA*, and *Lhcb1*), and promoted growth of rice in Cd-contaminated soil [85]. Therefore, by concurrently boosting nutrient uptake and enhancing photosynthetic efficiency, NFs implement a synergistic strategy that not only mitigates HMs toxicity but also fortifies the plant’s overall resilience and growth, Figure 4.

**Table 1:** Overview of nano-enabled agrochemicals for heavy metal remediation in agriculture.

Nanoparticles	Plant Species	Heavy Metals	Application Concentration	Impact on Plants	References
ZnO	Pea (*Pisum sativum* L.)	As	100–200–300–400 mg L^−1^	Improved growth, antioxidative system, reduced oxidative stress, As uptake, and increased yield	[86]
ZnO	Maize (*Zea mays* L.)	Cd	25–50 mg L^−1^	Improved chlorophyll pigments and enhanced the activity of antioxidant enzymes and decreased oxidative stress	[87]
ZnO	Wheat (*Triticum aestivum*)	Cd	300 mg kg^−1^	Increased growth attributes (root, shoot, husk, and grains dry weight) by alleviating Cd concentration in plants	[88]
ZnO	*Solanum lycopersicum* L.	Cd	50 mg L^−1^	The NPs help in maintaining photosynthesis efficiency and enhancing the plant’s antioxidant defense, which includes enzymes like SOD and CAT, which reduce oxidative damage	[89]
ZnO	Soybean (*Glycine max*)	As	50–100 mg L^−1^	Enhanced growth, increased photosynthetic pigments, antioxidant enzymes, and reduced ROS	[90]
ZnO	Rice (*Oryza sativa* L.)	As	10–100 mg L^−1^	Increased growth, phytochelatin content, anti-oxidative system, and decreased As accumulation	[91]
SiO_2_	Wheat (*Triticum aes-tivum*)	Cd	300–600 mg L^−1^	Decreased oxidative stress, increased photosynthesis, antioxidant enzyme activities, reduced Cd concentrations in tissues, grains, and enhanced Si contents in plants	[79]
SiO_2_	Soybean (*Glycine max*)	Hg	500 mg L^−1^	Increased growth, chlorophyll pigments, reduced Hg uptake and accumulation in both tissues	[92]
SiO	*Brassica napus* L.	Cd	250 mg kg^−1^	Increased biomass, chlorophyll pigments, carotenoids, photosynthetic rate, decreased cellular oxidative stress by improving antioxidative system and Cd translocation from root to shoot	[52]
SiO	Tomato (*Solanum lycopersicum* L.)	As	250, 1000 mg L^−1^	Increased growth, decreased As uptake, translocation and mitigated phytotoxicity, increased yield	[93]
FeO	Rice (*Oryza sativa* L.)	Cd	10–15 mg L^−1^	Enhanced growth, nutrient uptake photosynthetic parameters, and reduced oxidative stress by improving stress-responsive genetic mechanism	[94]
FeO	Wheat (*Triticum aestivum*)	Cd	100 mg kg^−1^	Immobilized Cd in soil, increased photosynthesis, nutrient uptake, growth, and enhanced antioxidative system, decreased oxidative stress	[34]
Fe_3_O_4_	Coriander (*Coriandrum sativum*)	Cd, Pb	100 mg L^−1^	Improved growth, reduced uptake of Cd and Pb and oxidative stress	[95]
Fe	Wheat (*Triticum aestivum* L.)	Cd	10 mg L^−1^	Increased Fe uptake, plant growth parameters, photosynthesis, and strengthened antioxidative system	[96]
Ti	*Vigna radiata* L.	As	10–50 mg L^−1^	Increased growth and biomass, enhanced antioxidant activities, induced stress-responsive genes, decreased ROS	[71]
TiO_2_	*Glycine max*	Cd	100–300 mg kg^−1^	Immobilized Cd in soil, reduced uptake, increased chlorophyll and net photosynthesis, RWC, growth parameters, reduced lipid peroxidation	[97]
TiO_2_	Coriander (*Coriandrum sativum* L.)	Cd	80 mg L^−1^	Improved growth, chlorophyll pigments, photosynthetic rate, transpiration rate, stomatal conductance, total soluble sugars, and antioxidants enzymes activities	[98]
Ag	*Moringa oleifera*	Cd, Pb	200 mg kg^−1^	Immobilized Cd and Pb in soil, reduced their uptake, oxidative stress by increasing antioxidant activities	[99]
Ag	*Lupinus luteus* L.	Cd, Pb, Zn, Ni	25 mg kg^−1^	Improved growth, photosynthesis, GPX activity, and metallothioneins expression	[100]

### 3.4. Antioxidant Defense System Activation

Under HMs stress conditions, cellular redox homeostasis is usually disrupted, leading to the excessive accumulation of reactive oxygen species (ROS) [101]. These ROS can damage biomolecules such as lipids, proteins, and DNA, causing oxidative stress, ultimately inhibiting crop growth and yield [51]. To combat ROS, plants activate sophisticated defense systems comprising both enzymatic and non-enzymatic components [8]. Key enzymatic antioxidants, including superoxide dismutase (SOD), catalase (CAT), ascorbate peroxidase (APX), glutathione peroxidase (GPX), and glutathione reductase (GR), function synergistically to neutralize ROS and maintain cellular homeostasis [102].

Various studies have shown that applying ROS-scavenging NFs or nanozymes has been shown to mitigate HMs stress by activating antioxidative enzymes and enhancing plant resilience [4,22]. For instance, addition of Si-NPs (20 mg L^−1^) and Fe-NPs (10 mg L^−1^) increased SOD (90 and 72%) and CAT (50 and 71%), while decreasing MDA content by 20 and 50% in *Phaseolus vulgaris* plants subjected to Cd stress [76], Table 1. Similarly, foliar application of ZnO NPs (100 mg L^−1^) increased SOD (17%), CAT (31%), and POD (34%) activities, and decreased MDA levels by 61% in rice seedlings exposed to 100 μM Cr [103]. In wheat (*Triticum aestivum* L.) seedlings exposed to 500 µM Cu^2+^, the application of Si-NPs (2.5 mM) enhanced the activities of SOD (77%), POD (141%), CAT (68%), and APX (80%), while decreasing MDA and H_2_O_2_ contents by 31% and 19%, respectively [104].

Beyond direct enzymatic activation, NFs can enhance the antioxidant system by regulation of stress-responsive gene expressions [101]. For example, application of sulfur NPs (300 mg L^−1^) upregulated the expression of antioxidant encoding genes *BnPOD*, *BnAPX*, and *BnGST* (Glutathione S-transferase), thereby reducing oxidative stress in *Brassica napus* L. under Hg toxicity [105]. Likewise, the addition of Se-NPs (20 mg L^−1^) decreased the production of H_2_O_2_ by downregulating the expression of NADPH oxidases (*BnaRBOHF1*, *BnaRBOHC*, and *BnaRBOHD1*), thereby mitigating membrane lipid damage caused in *Brassica napus* L. plants under Cd stress [106], Table 1.

Furthermore, plants frequently increase their production of non-enzymatic antioxidants like glutathione (GSH) and ascorbic acid (ASA) in response to HMs stress [102]. For instance, exposure to Cd raises the ASA level in soybean, increasing the plant’s antioxidant capacity [90]. The addition of 25 mM CaO NPs increased the activities of SOD (38%), CAT (37%), APX (15%), and GR (28%), elevated ASA (19%) and GSH (36%) levels, and reduced MDA (36%) and H_2_O_2_ (30%) contents, thereby alleviating Cd-induced oxidative stress in barley seedlings [81]. A central component of this defense is the ascorbate-glutathione (Asc-Glu) cycle, in which enzymes like APX and GR work to reduce H_2_O_2_ to H_2_O, thereby preventing oxidative damage [101]. Supporting this, application of ZnO-NP (100 mg L^−1^) increased the activities of APX (89%), GSH (21%), and GR (39%), while decreasing MDA (22%) and H_2_O_2_ (128%) levels in As-stressed soybean plants [90]. A key antioxidant, GSH, detoxifies ROS directly and acts as a substrate for GPX. Synthesized from GSH, phytochelatins (PCs) help to reduce the toxicity of HMs by chelating and helping to sequester them into vacuoles [8]. The application of Si-(100 mg L^−1^) and TiO_2_-NPs (50 mg L^−1^) upregulated the expressions of *ABC1* (3.3- and 2.1-fold), PCS (4.5- and 3.2-fold), and *GSH1* gene, which increased GSH and PC’s accumulation in roots and shoots, and insured As transportation to the vacuoles in *Oryza sativa* L. under As stress [68]. Consequently, this mechanism shielded the leaves from oxidative damage and enhanced the photosynthetic system and growth of the rice plants.

### 3.5. Effects of HMs on Protein, Osmolytes, and Synthesis of Signaling Molecules

Nano-agrochemicals play a regulatory role in plant responses to HM stress by modulating osmolyte synthesis and maintaining hormonal balance, thereby enhancing plants resilience [35], Figure 4. Nano-agrochemicals serve as a protective mechanism, helping to maintain physiological and biochemical stability under HM toxicity [20,35]. Key osmolytes such as proline, glycine betaine (GB), and soluble sugars (SS) are synthesized in greater amounts to mitigate HM-induced stress. For instance, ZnO-NPs increased proline and GB synthesis by 83% and 72%, respectively, while reducing MDA, H_2_O_2_, and EL by 69%, 97%, and 72% in *solanum lycopersicum* L., improving water balance and Cd tolerance [107]. Similarly, combined application of ZnO- and Se-NPs, both at a concentration of (25 μM L^−1^), markedly increased proline and GB levels in roots (264%, 135%), and (213%, 234%), and in shoots (172%, 105%), and (157%, 134%), respectively, which enhanced growth and resilience of *Glycine max* L. against As toxicity [108]. Foliar application of ZnO-NPs (100 mg L^−1^) also enhanced proline (158%), GB (92%), and SS (46%) in alfalfa (*Medicago sativa* L.), reinforcing Cd tolerance [109]. These findings indicate that nano-agrochemicals help restore osmotic homeostasis through osmolyte accumulation, bolstering plant resilience under HM stress.

Amino acids serve as precursors for secondary metabolites such as flavonoids and phenolics, which contribute to HM detoxifying and oxidative stress reduction [110]. For example, ZnO-NPs (20 mg kg^−1^) increased the synthesis of anthocyanins, flavonoids, and total phenols in Cucumis melo, improving its antioxidative defense and resilience to Cd stress [111]. These metabolites chelate HM ions and scavenge free radicals, aiding in detoxification and cellular homeostasis [110]. Phenylalanine ammonia-lyase (PAL) plays a critical role in phenylalanine lignification, strengthening physical barriers and reducing HM entry to cell. Addition of ZnO-NPs (5 mg L^−1^) enhanced PAL activity in roots/leaves (4/11%) and reduced phenylalanine concentrations in root/leaves (17/5%) of *Lactuca sativa* L., which reduced Cd absorption in plants [108].

Despite these advances, significant gaps remain in understanding the mechanisms by which nano-agrochemicals stimulate the synthesis of these protective compounds under HM stress. Further research is needed to elucidate the complex interactions between nano-agrochemicals and signaling molecules, which will inform targeted strategies to enhance plant resilience and productivity in contaminated environments.

### 3.6. Activation of Phytohormone and Antioxidant Enzymes Pathways

The activation of phytohormone and antioxidant enzyme pathways is a crucial mechanism by which nano-agrochemicals enhance plant resilience to HM stress. Phytohormones including gibberellins (GAs), abscisic acid (ABA), ethylene (ET), auxins (IAAs), isopentenyl adenosine (IPA), cytokinins (CKs), brassinosteroids (BRs), and jasmonic acid (JA) are important not only for plant growth but also for remediation, as they trigger signaling pathways that improve the plant’s capacity to detoxify HMs [22]. Nano-agrochemicals have the ability to alter the levels of endogenous phytohormones to enhance stress resilience in plants [103]. Specifically, phytohormones including IAA, GA, and ABA control the genes expressions involved in HMs transport and sequestration, a process that enables plants to bind HMs to the cell wall or compartmentalize in vacuoles, thereby lowering their toxicity [10]. For instance, the addition of nZVI (1000 mg L^−1^) markedly upregulated ABA and IPA contents by 45% and 16%, respectively, increasing the stress resilience of rice plants under Cd stress [93]. Similarly, application of ZnO-NPs (50 μM L^−1^) significantly increased ABA (118%), IAA (149%), JA (92%), and GA content (160%) in *Glycine max* L. under As stress [112]. Furthermore, hormones like ET and BRs influence the production of PCs, which bind to HMs and facilitate their vacuolar sequestration to reduce the HMs load in critical tissues [113]. This was demonstrated when application of ZnO-NPs (100 mg L^−1^) markedly increased BRs (2.08-fold) and PCs contents, thereby decreasing Cr concentrations in rice tissues [103]. These hormonal changes are closely linked to the stimulation of antioxidant defenses. Signaling molecules like JA, ABA, and SA activate these responses [114]. For instance, application of Si-NPs (200 mg kg^−1^) markedly increased SA (13%) and ABA (14%) contents, followed by SOD (40%) and POD (56%), and reduced MDA (19%) and H_2_O_2_ (52%) in Cd-stressed *Myrica rubra* plants [4]. Likewise, the foliar application of Si-NPs (10 mg L^−1^) conferred improved growth and stress resilience to *Cucumis sativus* L. under Cd toxicity by regulating key phytohormones (IAA, ABA, GA, and JA) and strengthening the plant’s antioxidative system [115]. Through these interconnected mechanisms, phytohormones orchestrate a comprehensive defense, enhancing plant resilience to HM stress and facilitating growth in contaminated environments.

### 3.7. Activation of Stress-Responsive Transcription Factors

Transcription factors (TFs) are pivotal in gene expression, and increase plants’ resilience to HMs stress by activating downstream defense mechanisms [116]. A specific group of TFs, including bZIP, HSF, MADS, HEX, GATA, NAC, bHLH, and MYB, are differentially regulated under HM stress across various plant species and are essential for orchestrating adaptive responses [117]. For instance, the TFs (MYB and HSF) can modulate the antioxidant pool and suppress the expression of HM transport-related genes [118]. These TFs are essential for the growth and developmental process as well as tolerance to various environmental stimuli. Nano-agrochemicals have emerged as effective agents for modulating these TFs to confer tolerance [106]. In soybean (*Glycine max* L.) under As stress, Se-NPs increased the abundance of bHLH, MYB, HSFs, and bZIP TFs, which in turn activated genes crucial for stress resilience [117]. Similarly, in Cd-stressed *Brassica napus* L. plants, the application of Se-NPs (20 mg L^−1^) increased the transcription of *BnaMYB* and *BnabHLH*, which in turn maintained Ca^2+^ homeostasis and reduced oxidative protein and membrane lipid peroxidation [106]. This suggested that Se-NPs promote intracellular Ca^2+^ flux, leading to the downregulation of glycolate oxidase (*BnaGLO*) and NADPH oxidases (*BnaRBOHs*), thereby balancing ROS production and scavenging. Furthermore, a combined application of ZnO-NPs (50 μML^−1^) + SeNPs (10μML^−1^) upregulated the expression of WRKY TFs (*GmWRKY106*, *GmWRKY56*, *GmWRKY46*, and *GmWRKY6*) and downregulated the expressions of As uptake and transport-related genes (*GmPT8*, *GmPT4*, *GmPT3*, *GmPT2*, and *GmPT1*), which reduced the uptake and acropetal translocation of As in *Glycine max* L. plants [119]. Collectively, these findings demonstrate that nano-agrochemicals mitigate HMs uptake, translocation, and toxicity in plants by activating specific transcription factors. This mechanism positions them as promising, eco-friendly tools for enhancing crop growth and yield in contaminated soils.

## 4. Environmental Impacts and Safety Considerations

While the adoption of nano-agrochemicals over the past decade has undoubtedly benefited sustainable agriculture by improving nutrient use efficiency, soil health, crop production, and HM remediation, their deliberate use may also pose risks of irreversible environmental harm [120]. However, their application introduces potential risks of irreversible environmental harm due to the unique properties of engineered nanomaterials. The environmental behavior and impact of these nanomaterials are governed by their unique physicochemical properties and are influenced by both intrinsic factors (e.g., chemical composition) and external conditions (e.g., soil properties, temperature, pH) [121]. These interactions can lead to the aggregation of NPs in the rhizosphere, potentially causing instability, increased acidity, and toxicity that adversely affect both plants and soil symbionts [4]. A critical process is NP aggregation within the rhizosphere, driven by factors including van der Waals forces, ionic strength, and interactions with natural organic matter [35]. This aggregation increases particle size, reduces effective surface area, and decreases NP mobility in porous media, as demonstrated by the non-absorption of larger Ag-NP aggregates (≥10 nm) by *Cicer arietinum* L. roots [122]. These colloidal interactions are described by the DLVO theory in saturated systems, while the extended DLVO (XDLVO) framework, which incorporates hydrophobic and capillary forces, is more applicable to unsaturated agricultural soils [123].

The presence of NPs can significantly alter soil physicochemical properties, impacting its structure, aeration, and water retention capacity. For instance, metallic NPs have been shown to inhibit soil aggregation, leading to reduced porosity [19]. Furthermore, NPs are subject to vertical transport through the soil profile via water movement, posing a potential risk of groundwater contamination [124]. The extent of this transport is a function of NP characteristics (size, shape, surface chemistry) and soil parameters (porosity, moisture content) [125]. Concurrently, NPs undergo chemical transformations including oxidation, reduction, and dissolution, which alter their morphology, reactivity, and bioavailability. Their surface chemistry, including functional groups and coatings, is a dominant factor controlling these interactions and their ultimate environmental fate [35].

The biological impact of NPs on soil ecosystems is a primary concern. Nanoparticles can adversely affect microbial communities, thereby disrupting essential ecosystem services such as nutrient cycling and organic matter decomposition [30]. A key environmental concern is the pH-dependent dissolution of NPs like ZnO-NPs. In acidic soils, their rapid dissolution releases high Zn^2+^ ions, which can cause phytotoxicity, harm soil microbes, and cause pollution, ultimately counteracting their remediation benefits [19]. Specific studies report that AgNPs (100 mg kg^−1^) inhibit vital soil enzymes like arylamidase and phenol oxidase and alter the metabolic activity of plant-growth-promoting bacteria, affecting the synthesis of 1-aminocyclopropane-1-carboxylic acid deaminase, indole-3-acetic acid, and siderophores [126]. Similar impacts of CNTs including both (SWNTs and MWNTs) have been reported in reduction of ammonia-oxidizing microorganisms in soil [127]. Various other disadvantages of metal-based nano-agrochemicals such as Ag, Si, CeO_2_, TiO_2_, CuO, and ZnO on different crop species have been reported by [15], Table 2. These reported inhibitory impacts include decreased seed germination, root/shoot length, photosynthetic rates, stunted plant growth, increased stress responses, necrosis, ROS, disruption of cell membranes, disturb cellular homeostasis, and interference with signal transduction [128]. The severity of these negative impacts is highly dependent on factors such as NP dosage, concentration, application method, plant species, and soil dynamics [4,129]. Consequently, further research is imperative to elucidate NP dose–response relationships, motility, transformation pathways, interactions with HMs, and mechanisms of uptake and accumulation in plants to optimize their safe and beneficial application in agriculture.

### 4.1. Risk Assessment and Ecotoxicological Profiling

**Figure 5:**
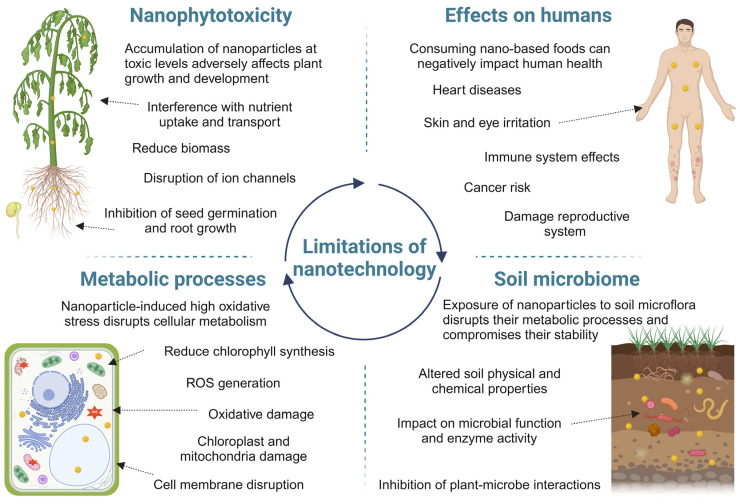
Overview of the limitations of nano-agrochemicals in the environment. Challenges include potential phytotoxicity, where excessive NPs concentrations disrupt plant growth and metabolic functions, and negative impacts on soil microbial communities, leading to reduced biodiversity and altered nutrient cycling. Nanoparticles may also pose detrimental effects on human beings such as immune and reproductive system damage.

Recent advancements in sustainable agriculture have undeniably demonstrated the successful use of certain nano-agrochemicals to enhance crop productivity [141]. However, various nano-agrochemicals can adversely affect soil properties, microbial communities, and plants, as well as human health, Figure 5. These potential impacts necessitate a robust and proactive risk assessment framework. The high reactivity of NPs, stemming from their small size and large specific surface area, raises significant safety concerns, particularly regarding occupational exposure for farm workers [141]. Therefore, the development of regulatory frameworks for nano-agrochemicals must be guided by ongoing research and comprehensive risk evaluations to ensure its safe implementation across sectors [142].

A critical pathway for ecosystem-level impact is the trophic transfer of NPs. Invertebrates and plants can absorb specific NPs, leading to bioaccumulation and potential translocation through the food chain. Nano-agrochemicals can disrupt the plant-associated microbiome including the rhizosphere, rhizoplane, and phyllosphere and impede essential microbial processes such as nitrogen fixation, phosphate solubilization, and nitrification [143]. Risk assessment must therefore quantify exposure pathways, including the environmental concentration, release kinetics, and persistence of NPs in soil [142,144]. Research indicates that the uptake, transformation, and bioaccumulation of NPs exhibit significant species-specific toxicities across different soil organisms and plants [145]. Beyond physiological effects, certain nano-agrochemicals (e.g., ZnO, AgO, TiO_2_, CNTs) can induce genotoxicity by interacting with cellular nucleic acids, leading to base alterations, chromosomal aberrations, dysregulated gene expression, and the suppression of DNA repair mechanisms [142]. Furthermore, associated human toxicities have been documented, affecting the respiratory, nervous, immune, endocrine, and reproductive systems, alongside carcinogenic potential [144,146]. Understanding the potential negative impacts of nano-agrochemicals necessitates ecotoxicological investigations using standardized testing techniques for accurate risk assessment [143].

### 4.2. Challenges in Scale-Up and Field Application: Bridging the Lab-to-Field Gap

While laboratory and greenhouse studies have well-documented the efficacy of nano-agrochemicals for HM remediation, their widespread adoption is hindered by significant challenges in scale-up and practical field application [144]. A critical barrier is the transition from gram-scale synthesis in research settings to the ton-scale production required for agricultural remediation. Many bottom-up synthesis methods for MONs, carbon nanomaterials, and polymeric nanocomposites involve complex procedures, high-energy inputs, and expensive precursors, making them economically unviable for large-scale agricultural use [147]. Furthermore, ensuring the batch-to-batch consistency, stability, and desired physicochemical properties (e.g., size, morphology, surface charge) of NPs during mass production remains a formidable technical challenge.

The application method itself presents another major challenge, often framed as the in situ versus ex situ remediation dilemma. Ex situ applications, where contaminated soil is excavated and treated in a controlled facility, allow for optimal NPs–soil contact and reaction conditions. However, this approach is prohibitively expensive, disruptive, and impractical for vast agricultural lands [128]. On the contrary, in situ application, directly applying nano-agrochemicals to the field, is the more feasible option for agriculture, but introduces immense complexity. Factors such as soil heterogeneity, organic matter content, pH variations, and microbial activity can lead to unpredictable NPs aggregation, transformation, and reduced mobility, thereby diminishing their remediation efficiency [19]. The potential for off-target movement of NPs to groundwater or adjacent ecosystems also necessitates rigorous monitoring during in situ applications, adding to the cost and management burden.

This contrast explains why traditional remediation techniques, such as vitrification, soil washing, and certain bioremediation strategies, continue to be employed despite their limitations [147,148]. These methods are built on established engineering principles with predictable costs and outcomes at large scales. Their operational protocols, machinery, and risk assessments are well-defined, making them a “known quantity” for project planners, even if they are less efficient or more disruptive than potential nano-solutions.

Therefore, future research must turn towards overcoming these scale-up and application barriers. This includes developing low-cost, green synthesis routes using agricultural waste, formulating nano-agrochemicals with coatings or composites that enhance their stability and targeted delivery in diverse soil types, and conducting long-term, large-scale field trials to validate their efficiency, economic feasibility, and environmental safety under real-world conditions.

## 5. Future Perspectives

To fully realize the potential of nano-agrochemicals while ensuring environmental safety, future research on nano-agrochemicals must focus on the development of novel nanocomposites, with better morphologies and functionalities for better outputs. In addition, the exploration of hybrid and multifunctional metallic NPs will open up new opportunities for advanced nanomaterials [120].

Integrating machine learning approaches can also accelerate the discovery and optimization of these NPs for various applications. By analyzing complex datasets encompassing NPs properties, soil chemistry, and plant physiology, predictive models can forecast efficacy, toxicity, and environmental fate. This will enable the rapid virtual screening and rational design of next-generation formulations, dramatically accelerating the optimization process.

The segregation of microbe-assisted nano-agrochemicals represents a highly promising strategy for the remediation of HMs in soils and the promotion of sustainable crop production [149]. Future research should focus on developing innovative nano-agrochemical formulations that synergize beneficial microorganisms with NPs to enhance the efficiency of HMs uptake and detoxification. These formulations can leverage the high surface area and reactivity of nanomaterials to enhance microbial activity and facilitate the immobilization or biotransformation of HMs, thereby promoting sustainable crop production.

Translating laboratory success to field application necessitates the development of comprehensive safety guidelines and regulatory frameworks. This requires interdisciplinary collaboration among nanotechnologists, agronomists, microbiologists, and environmental scientists to assess long-term environmental fate and ecotoxicological impacts. Such efforts are fundamental to facilitating the safe, effective, and publicly accepted adoption of nano-agrochemicals for sustainable agriculture.

## 6. Conclusions

Nano-agrochemicals undoubtedly represent a transformative approach for enhancing soil health, promoting crop growth, and remediating HMs contamination in agricultural systems. Their proven efficacy stems from unique physicochemical properties, such as high surface area and reactive functional groups, which effectively enable HM immobilization through mechanisms including adsorption, complexation, precipitation, and catalytic degradation. Within plants, these nano-agrochemicals decisively mitigate HM toxicity by downregulating metal transporter genes, enhancing antioxidant defenses to scavenge ROS, and improving nutrient delivery, thereby robustly maintaining cellular homeostasis. This powerful synergy between nanotechnology and plant biology offers a sustainable pathway to ensure food security and ecosystem health. The potential for eco-friendly and cost-effective application is significant and clear. To realize this potential on a widespread scale, further work must solidly hinge on optimizing nano-agrochemical formulations for maximum efficacy and minimal environmental impact, and on comprehensively evaluating their long-term performance across diverse soil types and plant species.

## Figures and Tables

**Table 2:** Negative impacts of nano-agrochemicals on plants.

Nanoparticles	Plant Species	Concentration	Negative Effects on Plants	References
ZnO NPs	*Phytolacca americana* L.	500 mg kg^−1^	Reduced root and shoot growth, increased lipid peroxidation, and severely damaged root cells	[130]
Cu NPs, ZnO NPs	Mung bean (*Vigna radiata* L.)	1000, 2000 mg L^−1^	Inhibited seed germination, embryo growth, disturbed micronutrients (Fe, Mn, Cu, Zn, K) and macronutrients (Ca, Na, Mg,), increased antioxidants	[131]
CuO	Lettuce	40 μg mL^−1^	Decreased seed germination and reduced radicle growth	[132]
NiO_2_	Wheat	120 mg kg^−1^	Reduced plant growth, inhibited photosynthesis, and increased antioxidant activities	[133]
NiO NPs	Chinese cabbage	50, 250, and 500 mg L^−1^	Reduced root growth, chlorophyll content, and carotenoid, increased lipid peroxidation and ROS production, caused molecular and metabolic changes	[134]
SiO_2_	Maize	1000 mg L^−1^	Adversely affected early growth parameters, reduced chlorophyll and carotenoid pigments, and triggered oxidative stress	[135]
TiO_2_ NPs	Barley (*Hordeum vulgare*)	2000 mg kg^−1^	Decreased biomass, photosynthesis, increased antioxidants (SOD, CAT), and induced oxidative stress	[136]
TiO_2_	Basil (*Ocimum basilicum*).	750 mg kg^−1^	Decreased chlorophyll b (52%), total chlorophyll (30%), reduced Mg contents in root by 115%	[137]
AgNPs	*Vicia faba*	100 mg L^−1^	Reduced photochemical efficacy of photosystem II (PSII), increased ROS	[138]
MWCNTs	*Cucurbita pepo* L.	125, 250, 500 mg L^−1^	Decreased germination percentage, shoot growth, biomass, increased oxidative damage.	[139]
GO NPs	Faba bean (*Vicia faba* L.)	100, 200, 400, 800, 1600 mg L^−1^	Reduced growth, CAT, and APX activity, increased electrolyte leakage	[140]
AgNP	*Brassica nigra*	200, 400, 800, 1600 mg L^−1^	Decreased soluble sugars, reduced sugars, inhibited seed germinating and seedling growth. Inhibited stress-responsive signaling pathways and key metabolic enzymes	[129]

## Data Availability

No new data were created or analyzed in this study.
